# Modulating Oxidative Stress in Drug-Induced Injury and Metabolic Disorders: The Role of Natural and Synthetic Antioxidants

**DOI:** 10.1155/2019/3206401

**Published:** 2019-06-27

**Authors:** Ayman M. Mahmoud, Fiona L. Wilkinson, Mansur A. Sandhu, Julia M. Dos Santos, M. Yvonne Alexander

**Affiliations:** ^1^Physiology Division, Zoology Department, Faculty of Science, Beni-Suef University, Beni-Suef, Egypt; ^2^Cardiovascular Science, The Centre for Bioscience, Faculty of Science and Engineering, Manchester Metropolitan University, Manchester, UK; ^3^Depertment of Biomedical Sciences, Faculty of Veterinary & Animal Sciences, PMAS, Arid Agriculture University, Rawalpindi, Pakistan; ^4^Detroit R&D Inc., Detroit, USA; ^5^School of Education, Health and Human Performance, Fairmont State University, West Virginia, USA; ^6^Manchester Academic Health Science Centre, Manchester, UK

Oxidative stress is an imbalance in cellular redox reactions which plays a key role in the pathogenesis of metabolic disorders and drug-induced injury [1–3]. Oxidative stress is the result of reactive oxygen species (ROS) overproduction or a decline in antioxidant defense mechanisms. Although ROS production can be beneficial in some instances as they are used by the immune system, in general, excessive generation of ROS results in deleterious effects causing damage to DNA, proteins, and lipids, ultimately leading to cell death. Several diseases, including cancer, neurodegeneration, obesity, metabolic syndrome, diabetes mellitus, and liver disease, are well-known to be associated with excessive ROS production. Therefore, agents counteracting excess ROS and/or boosting the antioxidant defenses represent an appealing strategy for the treatment of multiple diseases.

Antioxidant substances could be natural or synthetic. Natural antioxidants are obtained entirely from natural sources and have been used in food, cosmetics, and pharmaceutical industries. On the other hand, synthetic antioxidants are substances created from chemical processes. The current understanding of the complex role of ROS in the physiological and pathological processes points to the necessity of developing multifunctional antioxidants, which can maintain oxidative homeostasis, both in health and in disease. In this context, numerous research groups focus on the characterization and application of natural antioxidant agents in different diseases. In addition, a great deal of effort is being conducted to design and synthesize free radical-scavenging and antioxidant substances that can diminish excessive ROS production and improve the endogenous antioxidant defenses. In addition, reduction of ROS by either natural or synthetic agents has been associated with the attenuation of various diseases, including endothelial dysfunction [4, 5], diabetic cardiomyopathy [6], nephropathy [7], retinopathy [8] and gonadal dysfunction [9], carcinogenesis [10, 11], hyperammonemia [12], chronic subclinical systemic inflammation [13], fibrosis [2], and drug-induced toxicity [14, 15]. Various studies have attributed the reduction of ROS and oxidative stress as a direct consequence of nuclear factor erythroid 2-related factor 2 (Nrf2) signaling activation [1, 3–5, 10, 13, 14]. Understanding and validating the biological activities of natural and synthetic antioxidant compounds and their molecular mechanisms in counteracting ROS and oxidative stress will provide solid scientific foundation to the application of antioxidants in the prevention and treatment of multiple diseases.

This special issue encompasses 20 research articles focusing on the role of natural and synthetic antioxidants in ameliorating diseases associated with oxidative stress, such as diabetic cardiomyopathy, endothelial dysfunction, heat stress, pancreatic fibrosis, nonalcoholic steatohepatitis, sepsis, vascular inflammation, and peripheral neuropathy. In addition, the issue includes 3 review articles discussing recent findings in the role of antioxidants in renal replacement therapy and cardiovascular health and ROS-mediated epigenetic changes in radiation-induced fibrosis. The guest editors are pleased to present a compendium of these cutting-edge original research and review articles as follows.

In the research article “Anti-Inflammatory, Immunomodulatory, and Antioxidant Activities of Allicin, Norfloxacin, or Their Combination against *Pasteurella multocida* Infection in Male New Zealand Rabbits,” R. T. M. Alam et al. investigated the anti-inflammatory, antioxidant, and immunomodulatory effect of norfloxacin and allicin, an active constituent of *Allium sativum*, in rabbits infected with *Pasteurella multocida*. Treatment of the *Pasteurella multocida*-infected rabbits with allicin and/or norfloxacin ameliorated inflammation and oxidative stress.

In the research article “Protective Effect of Rosamultin against H_2_O_2_-Induced Oxidative Stress and Apoptosis in H9c2 Cardiomyocytes,” L. Zhang et al. examined the protective effect of rosamultin against hydrogen peroxide- (H_2_O_2_-) induced oxidative stress and apoptosis in H9c2 cardiomyocytes. The results showed that pretreatment of the H_2_O_2_-induced cardiomyocytes with rosamultin reversed the morphological changes; reduced the release of lactate dehydrogenase and creatine kinase, production of ROS, and lipid peroxidation; and suppressed the expression of proapoptotic mediators. In addition, rosamultin boosted antioxidant enzymes and enhanced the expression of antiapoptotic mediators in H_2_O_2_-induced cardiomyocytes.

In the research article “Growth Inhibition of a Novel Iron Chelator, DpdtC, against Hepatoma Carcinoma Cell Lines Partly Attributed to Ferritinophagy-Mediated Lysosomal ROS Generation,” an interesting study conducted by T. Huang et al., the link between ferritinophagy and the anticancer activity of the iron chelator, di-2-pyridylketone dithiocarbamate (DpdtC), has been studied. DpdtC mobilized iron from ferritin and exhibited antitumor effect against the hepatoma cell lines HepG2 and Bel-7402. According to the results, ferritinophagy-mediated lysosomal ROS generation was assumed to play a key role in the antiproliferative action of DpdtC.

In the research article “Curcumin Inhibits Acute Vascular Inflammation through the Activation of Heme Oxygenase-1,” Y. Xiao et al. investigated the protective effect of curcumin against acute vascular inflammation, pointing to the role of heme oxygenase 1 (HO-1). New Zealand white rabbits were fed a diet supplemented with 0.3% curcumin, and acute vascular inflammation was induced by putting a collar on the left common carotid artery for 24 h. In addition, HO-1 inhibitor and siRNA were used to study the involvement of HO-1 in mediating the effects of curcumin. Rabbits supplemented with dietary curcumin showed increased serum bilirubin and vascular, hepatic, and splenic HO-1 expression and decreased vascular inflammation, effects that were abolished by pharmacological- and siRNA-mediated inhibition of HO-1. These finding were supported by increased Nrf2 and HO-1 expression in endothelial cells treated with curcumin *in vitro*.

In the research article “Novel Curcumin C66 That Protects Diabetes-Induced Aortic Damage Was Associated with Suppressing JNK2 and Upregulating Nrf2 Expression and Function,” the protective effect of the curcumin analogue C66 on diabetes-induced aortic damage was introduced by C. Li et al. Diabetes was induced in male JNK2^−/−^ mice with a single intraperitoneal injection of streptozotocin (STZ). Both diabetic and control mice were treated with C66 for three months and samples were collected for analyses. C66 treatment as well as JNK2 deletion reversed diabetes-induced aortic oxidative stress, apoptosis, inflammation, and apoptosis and activated Nrf2. However, C66 showed no extra effect on Nrf2 and diabetic aortic damage without JNK2. Therefore, the protective effect of C66 was exerted mainly *via* inhibition of JNK2 accompanied with upregulation of Nrf2.

In the research articles “*Monolluma quadrangula* Protects against Oxidative Stress and Modulates LDL Receptor and Fatty Acid Synthase Gene Expression in Hypercholesterolemic Rats” and “Antidiabetic Effect of *Monolluma quadrangula* Is Mediated via Modulation of Glucose Metabolizing Enzymes, Antioxidant Defenses, and Adiponectin in Type 2 Diabetic Rats,” M. N. Bin-Jumah has provided two studies showing the beneficial effects of *Monolluma quadrangula* extract in hypercholesterolemic and diabetic rats. *Monolluma quadrangula* modulated the expression of LDL receptor and fatty acid synthase and protected rats against oxidative stress induced by hypercholesterolemic diet. In high-fat diet (HFD)/STZ-induced type 2 diabetic rats, *Monolluma quadrangula* extract improved glucose tolerance and reduced serum lipids, lipid peroxidation, and proinflammatory cytokines. In addition, *Monolluma quadrangula* modulated glucose metabolizing enzymes and increased both serum levels and hepatic expression of adiponectin.

In the research article “Thymoquinone Attenuates Cardiomyopathy in Streptozotocin-Treated Diabetic Rats,” in a rat model of diabetic cardiomyopathy, M. S. Atta et al. investigated the therapeutic potential of thymoquinone, the active constituent of *Nigella sativa* seeds. Diabetes was induced by STZ and diabetic rats received 50 mg/kg thymoquinone for 12 weeks. Treatment with thymoquinone ameliorated the cardiac expression of inducible nitric oxide synthase (iNOS) and oxidative stress markers and decreased serum lipids and inflammatory mediators.

In the research article “Parenteral Succinate Reduces Systemic ROS Production in Septic Rats, but It Does Not Reduce Creatinine Levels,” by using rats with cecal ligation and puncture as model of sepsis, S. P. Chapela et al. investigated whether parenteral succinate reduces systemic ROS production and improves kidney function. The results showed that succinate treatment of the rats subjected to cecal puncture reduced systemic ROS levels, whereas circulating creatinine levels were not affected.

In the research article “Camalexin Induces Apoptosis via the ROS-ER Stress-Mitochondrial Apoptosis Pathway in AML Cells,” camalexin is a phytoalexin with potent antitumor properties. It accumulates in various cruciferous plants upon exposure to plant pathogens and environmental stress. Y. Yang et al. aimed to investigate the effects of camalexin on human leukemic cells (AML cells). Camalexin suppressed the viability of leukemic cells and induced apoptosis *via* the mitochondrial pathway in a caspase-dependent manner. Upstream of apoptosis, camalexin induced endoplasmic reticulum (ER) stress. In addition, camalexin increased ROS, superoxide dismutase, and catalase, while glutathione was declined in AML cells. Furthermore, the administration of camalexin suppresses xenograft tumor graft growth without obvious toxicity.

In the research article “Optimization of Experimental Settings for the Assessment of Reactive Oxygen Species Production by Human Blood,” an interesting research article, T. Soares et al. provided a protocol to optimize the experimental conditions for the detection of ROS produced by human blood from healthy donors following stimulation with the potent inflammatory mediator phorbol-12-myristate-13-acetate (PMA). In their experiment, the probes fluorescent 2,7-dichlorodihydrofluorescein diacetate (DCFH-DA), 2-[6-(4-amino)-phenoxy-3H-xanthen-3-on-9-yl] benzoic acid (APF), and 10-acetyl-3,7-dihydroxyphenoxazine (amplex red) have been used. The results of this study can help researchers to select the accurate experimental conditions for their experiments, mimicking the unexplored *in vivo* settings by using a physiological *in vitro* system, and to save time and money.

In the research article “Coenzyme Q10 Ameliorates Pancreatic Fibrosis via the ROS-Triggered mTOR Signaling Pathway,” in a mouse model of chronic pancreatitis (CP), R. Xue et al. studied the ameliorative effect of coenzyme Q10. The result revealed that both pretreatment and posttreatment of the CP mice with coenzyme Q10 decreased autophagy, activation of pancreatic stellate cells (PSCs), oxidative stress, histological changes, and collagen deposition. *In vitro*, treatment of the primary PSCs with coenzyme Q10 increased the expression levels of p-PI3K, p-AKT, and p-mTOR. Therefore, coenzyme Q10 alleviated pancreatic fibrosis by modulating the ROS-triggered PI3K/AKT/mTOR signaling pathway.

In the research article “Proanthocyanidins Antagonize Arsenic-Induced Oxidative Damage and Promote Arsenic Methylation through Activation of the Nrf2 Signaling Pathway,” M. Xu et al. investigated the effects of grape seed proanthocyanidin extract (GSPE) on oxidative damage and arsenic (As) methylation in L-02 human hepatocytes, pointing to the role of Nrf2. The results showed that GSPE antagonized AS-induced oxidative damage and methylation in human hepatocytes. Activation of Nrf2 signaling has been shown to mediate, at least in part, the protective effect of GSPE on AS toxicity.

In the research article “Indicaxanthin from *Opuntia ficus indica* (L. Mill) Inhibits Oxidized LDL-Mediated Human Endothelial Cell Dysfunction through Inhibition of NF-*κ*B Activation,” the potential of indicaxanthin from *Opuntia ficus indica* to prevent oxidized LDL-mediated endothelial dysfunction through inhibition of nuclear factor-kappaB (NF-*κ*B) activation has been investigated by A. Attanzio et al. Endothelial cell pretreated with 5 and 20 *μ*M indicaxanthin and incubated with oxidized LDL showed significantly declined expression of adhesion molecules and NF-*κ*B transcriptional activity. Thus, the use of nutritionally relevant concentrations of indicaxanthin protected endothelial cells against dysfunction induced by oxidized LDL.

In the research article “Alphalipoic Acid Prevents Oxidative Stress and Peripheral Neuropathy in Nab-Paclitaxel-Treated Rats through the Nrf2 Signalling Pathway,” nab-paclitaxel (nab-PTX) is an injectable formulation of paclitaxel (PTX) used to treat different types of cancer. The prevalence of peripheral neuropathy induced by nab-PTX has been reported to be higher than that induced by PTX. H. Sun et al. investigated the ability of alpha-lipoic acid (*α*-LA) to prevent nab-PTX-induced peripheral neuropathy in rats. *α*-LA inhibited oxidative stress and peripheral neuropathy in nab-PTX-administered rats by activating Nrf2 signaling pathway without diminishing the chemotherapeutic effect of nab-PTX.

In the research article “ROS Reduction Does Not Decrease the Anticancer Efficacy of X-Ray in Two Breast Cancer Cell Lines,” radiotherapy is a frequently administrated effective treatment for various types of cancer. The anticancer efficacy of X-ray radiotherapy has been frequently correlated with excessive generation of ROS. H. Wang and X. Zhang conducted this study to test the effect of reducing ROS levels on the antitumor efficacy of X-ray radiotherapy. The results showed that the use of ROS scavengers did not affect the X-ray-induced death of the breast cancer cell lines MDA-MB-231 and MCF-7.

In the Research article “In Vitro Anti-Inflammatory Effect of *Salvia sagittata* Ethanolic Extract on Primary Cultures of Porcine Aortic Endothelial Cells,” *Salvia sagittata* is used traditionally in Ecuador to treat inflammation and intestinal diseases. I. Tubon et al. investigated the effect of an ethanolic extract of *Salvia sagittata* (SSEE) on lipopolysaccharide- (LPS-) induced inflammation in primary cultures of porcine aortic endothelial cells (pAECs). Treatment of the pAECs with different concentrations of SSEE did not affect the cell viability, while it showed a remarkable ability to reduce LPS-induced production of proinflammatory cytokines and increase the expression of HO-1.

In the research article “Protective Effects of Inorganic and Organic Selenium on Heat Stress in Bovine Mammary Epithelial Cells,” Y. Zou et al. explored and compared the protective effects of inorganic selenium (sodium selenite, SS) and organic selenium (selenite methionine, SM) in mammary alveolar cells-large T antigen (MAC-T) and bovine mammary epithelial cell line (BMEC) during heat stress. Both SS and SM protected the cells against the heat shock-induced redox imbalance and cell death. SM was more effective in modulating the expression of Nrf2 and iNOS, whereas the protective effect of SS was associated with thioredoxin reductase 1.

In the research article “Glycine Suppresses AGE/RAGE Signaling Pathway and Subsequent Oxidative Stress by Restoring Glo1 Function in the Aorta of Diabetic Rats and in HUVECs,” the role of advanced glycation end product (AGE) accumulation in vascular damage has been well-acknowledged. In this context, Z. Wang et al. evaluated whether glycine, the simplest amino acid, can attenuate oxidative stress by suppressing the AGE/RAGE signaling pathway. The results showed that the oral administration of glycine increased nitric oxide (NO) content and ameliorated oxidative stress and attenuated AGE/RAGE signaling pathway in the aorta of diabetic rats. The ameliorative effect of glycine was associated with increased activity and expression of aortic glyoxalase-1 (Glo1). In methylglyoxal-induced endothelial cells, glycine suppressed ROS generation and AGE/RAGE signaling pathway.

In the research article “Simvastatin Reduces Hepatic Oxidative Stress and Endoplasmic Reticulum Stress in Nonalcoholic Steatohepatitis Experimental Model,” G. Rodrigues et al. investigated the efficacy of simvastatin, a lipid-lowering drug, to prevent methionine/choline-deficient diet-induced nonalcoholic steatohepatitis in mice. Treatment with simvastatin reduced liver injury, hepatic lipids, and hepatocellular ballooning. In addition, simvastatin ameliorated lipid peroxidation, inhibited endoplasmic reticulum stress, and boosted the antioxidant enzymes and Nrf2 expression.

In the review article “Antioxidant Supplementation in Renal Replacement Therapy Patients: Is There Evidence?,” end-stage renal disease patients, on hemodialysis or peritoneal dialysis, exhibit oxidative stress and increased risk for cardiovascular disease. In a review article, V. Liakopoulos et al. presented and discussed the available data regarding the exogenous administration of antioxidants and their possible protective effects on renal replacement therapy patients.

In the review article “Reactive Oxygen Species Drive Epigenetic Changes in Radiation-Induced Fibrosis,” S. Shrishrimal et al. highlighted the role of ROS-mediated epigenetic changes in radiation-induced fibrosis (RIF). The authors reviewed the ROS-mediated changes in metabolism, TGF-*β* signaling, DNA methylation, histone modification, and noncoding RNA changes in RIF.

In the review article “Beneficial Effects of Citrus Flavonoids on Cardiovascular and Metabolic Health,” A. M. Mahmoud et al. reviewed and discussed the recent findings and advances in understanding the mechanisms underlying the protective effects of citrus flavonoids against various diseases. The biological activities of citrus flavonoids in oxidative stress, lipid metabolism, and adipose tissue inflammation and their therapeutic potential in diabetes, diabetic cardiomyopathy, endothelial dysfunction, and atherosclerosis have been discussed. This review article pointed to the need of further studies and clinical trials to assess the efficacy and to explore the underlying mechanism(s) of action of citrus flavonoids.

The editors anticipate this special issue to be of interest to the readers and expect researchers to benefit in making further progress in the understanding of the role of natural and synthetic antioxidants in the treatment of various diseases.
